# Overexpression of Notch1 is associated with the progression of cervical cancer

**DOI:** 10.3892/ol.2020.12395

**Published:** 2020-12-18

**Authors:** Yan Sun, Rui Zhang, Shujuan Zhou, Yuqiang Ji

Oncol Lett 9: 2750-2756, 2015; DOI: 10.3892/ol.2015.3143

Subsequently to the publication of the above article, an interested reader drew to the authors' attention that, in Fig. 4D on p. 2754, the data shown in the siRNA-Ctr, SiHa and siRNA-Ctr, C33A panels appeared to be strikingly similar.

After having re-examined their data, the authors realized that Fig. 4D had been assembled incorrectly, and the data for the siRNA-Ctr, SiHa experiment had inadvertently been selected twice. The revised version of Fig. 4, showing the corrected data for the siRNA-Ctr, C33A experiment, is shown on the next page. Note that the error made in this Figure did not affect the results or the conclusions reported in this paper, and all the authors agree to this Corrigendum. The authors thank the Editor of *Oncology Letters* for presenting them with the opportunity to publish this Corrigendum, and apologize to the Editor and to the readership of the Journal for any inconvenience caused.

## Figures and Tables

**Figure 4.**
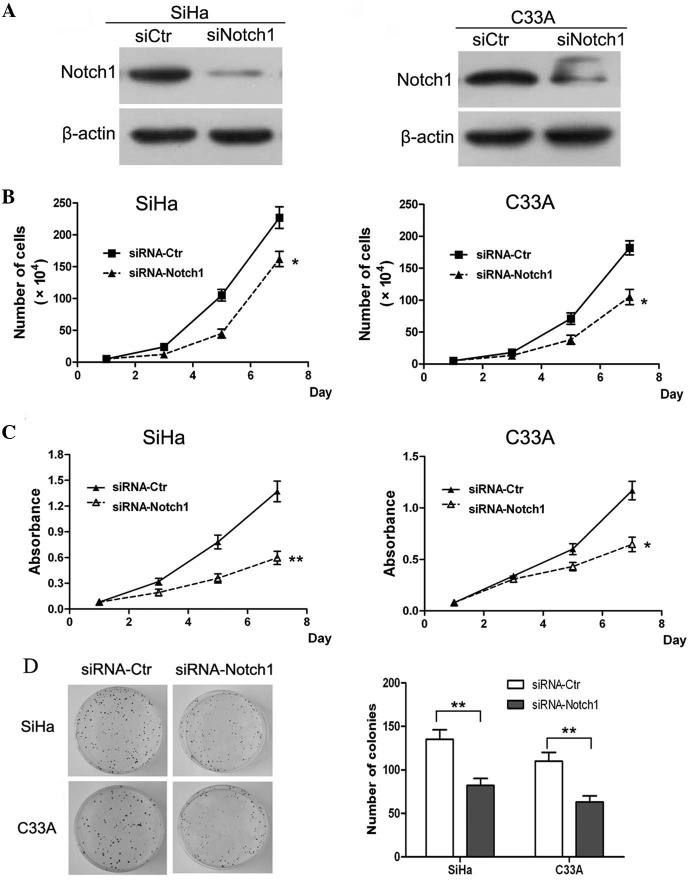
Knockdown of Notch1 inhibited the proliferation and colony formation of cervical cancer cells *in vitro*. SiHa and C33A cells were transfected with siRNA oligonucleotides and harvested following 24 h of incubation. (A) Western blotting revealed the knockdown of Notch1 in SiHa and C33A cells. (B) Knockdown of Notch1 inhibited the proliferation of SiHa and C33A cells*in vitro*. (C) Knockdown of Notch1 inhibited the viability of SiHa and C33A cells *in vitro*. (D) Knockdown of Notch1 inhibited the colony forming ability of SiHa and C33A cells. Results are presented as the mean ± standard deviation from three experiments performed in duplicate. *P<0.05 and **P<0.01 vs. control. siRNA, small interfering RNA; Ctr, control.

